# Dietary geraniol ameliorates intestinal dysbiosis and relieves symptoms in irritable bowel syndrome patients: a pilot study

**DOI:** 10.1186/s12906-018-2403-6

**Published:** 2018-12-19

**Authors:** Fernando Rizzello, Chiara Ricci, Michela Scandella, Elena Cavazza, Elisabetta Giovanardi, Maria Chiara Valerii, Massimo Campieri, Antonietta Comparone, Luigia De Fazio, Marco Candela, Silvia Turroni, Enzo Spisni

**Affiliations:** 1 Department of Medical and Surgical Sciences, University of Bologna, Via Massarenti 9, 40138 Bologna, Italy; 2 Department of Clinical and Experimental Sciences, University of Brescia, Spedali Civili 1, 25121 Brescia, Italy; 3 Department of Biological, Geological and Environmental Sciences, Biology Unit, University of Bologna, Via Selmi 3, 40126 Bologna, Italy; 4 Department of Pharmacy and Biotechnology, University of Bologna, Via Belmeloro 6, 40126 Bologna, Italy

**Keywords:** Geraniol, Irritable bowel syndrome (IBS), Microbiota, Inflammation, Dysbiosis

## Abstract

**Background:**

(Trans)-3,7-Dimethyl-2,6-octadien-1-ol, commonly called geraniol (Ge-OH), is an acyclic monoterpene alcohol with well-known anti-inflammatory and antimicrobial properties. Ge-OH is a non-toxic compound classified as Generally Recognized As Safe (GRAS) by the US Food and Drug Administration and the European Food Security Agency.

**Methods:**

Ge-OH was orally administered at a maximum daily dose of 8 mg kg^(− 1)^ body weight for four weeks in a delayed release formulation capable of reaching the colon. Fecal microbiota and blood cytokines were analyzed before and after Ge-OH treatment, as well as IBS symptomatology by using Visual Analogue Scale (VAS-IBS).

**Results:**

The results show that orally administered Ge-OH is a powerful modulator of the intestinal microbial ecosystem, capable of leading to increased relative abundances of *Collinsella* and especially *Faecalibacterium*, a well-known health-promoting butyrate producer consistently found to be decreased in IBS patients. Moreover, Ge-OH strongly improved the clinical symptoms of colitis by significantly reducing the score recorded by the VAS-IBS questionnaire. Clinical improvement was associated with a significant reduction in the circulating MIP-1β, a chemokine found to be increased in several IBS patients.

**Conclusion:**

Ge-OH could be a powerful component for food supplement targeted to the treatment of IBS patients.

**Trial registration:**

ISRCTN47041881, retrospectively registered on 19th July 2018.

## Background

Irritable bowel syndrome (IBS) affects 9–23% of the population across the world. It is considered a debilitating disease because it strongly impairs quality of life in those affected [1] and directly impacts the working segment causing a 21% loss in productivity [2]. Abdominal pain, discomfort and bloating are common symptoms in all affected patients, but the disorder can be classified as diarrhea-predominant (IBS-D), constipation-predominant (IBS-C) and alternating stool pattern (IBS-A) based on intestinal habit [3]. IBS is difficult to diagnose given the heterogeneity of symptoms and comorbidities that are often associated, such as gastro-esophageal reflux, non-celiac wheat sensitivity and fibromyalgia [4]. The most adopted criteria for diagnosis are the Rome III criteria, which state that a patient must have recurrent abdominal pain or discomfort at least three days/month in the last three months, associated with two or more of the following: improvement with defecation, onset associated with a change in stool frequency, onset associated with a change in form (appearance) of stools [5]. IBS therapy is mainly targeted to control patients’ symptomatology and includes low-dose antidepressants, spasmolytics and 5-HT3 antagonists. However, treatments are often not effective or not tolerated by patients [6]. Different mechanisms have been implicated in IBS pathogenesis, including altered gastrointestinal motility, visceral hypersensitivity and imbalanced cytokine signaling [7] that could involve IL-1β, IL-2, IL-4, IL-5, IL-6, IL-8, IL-10, IL-12, IL-17A, IFN-γ, TNF-α [7] and the chemokines MCP-1 and MIP-1β [8]. Even if none of them can be considered as a specific biomarker with a specific role in IBS pathogenesis, different studies indicate that a low-grade inflammation occurs in IBS patients [7, 8].

Several more recent studies have stressed the important relationship between gut microbiota dysbiosis and IBS [9]. Key findings in IBS dysbiosis include an increase in the Firmicutes to Bacteroidetes ratio, a decrease in the Lactobacilli and Bifidobacteria population, an increase in Streptococci and *Ruminococcus*, and a decrease of health-promoting butyrate-producing bacteria [10–12].

Probiotics and prebiotics have been investigated to evaluate their efficacy in improving symptoms in IBS patients. Systematic reviews and meta-analyses show that probiotics can significantly improve some symptoms in IBS patients [13]. However, the high variability among studies in terms of design, populations, probiotic strains and formulation used, weakens the evidence of their efficacy [14]. Prebiotics, such as inulin-type fructans and galacto-oligosaccharides, are able to modulate microbiota composition. In particular, inulin and oligofructose are known to have a bifidogenic effect. Few clinical trials have been conducted with prebiotics in IBS patients and the evidence of their efficacy is feeble [15]. In some patients, prebiotics improved the overall symptomatology but caused a worsening in bloating, and in one study prebiotic administration led to disease exacerbation, probably as a consequence of increased fermentative processes occurring in the colon [14]. Recently, the use of a wide-spectrum non-absorbable antibiotic for the treatment of IBS-associated dysbiosis has also been proposed [16].

Essential oil (EO) mixtures have been shown to play a significant role in the modulation of gut microbiota even if their mechanism(s) of action remain incompletely understood [17]. EOs have been recognized as potential new treatment options for IBS [18]. Geraniol (Ge-OH) is a naturally acyclic monoterpene component of EOs extracted from lemongrass, rose and other aromatic plants. Several studies on the biological activities of Ge-OH have shown it to be a highly active antimicrobial compound with antioxidant and anti-inflammatory properties [19, 20]. Ge-OH antimicrobial activities do not seem to have specific targets. Like other EOs, Ge-OH is a hydrophobic compound able to bind to the bacterial cell wall modifying its dynamic organization, with a consequent loss of ions and ATP depletion [21, 22]. In addition to bacterial growth inhibition, Ge-OH effectively modulates the drug resistance of several Gram-negative bacteria, such as *Enterobacter aerogenes*, *Escherichia coli* and *Pseudomonas aeruginosa*, by restoring drug susceptibility in strains overexpressing efflux pumps [23]. Moreover, human pathogenic bacteria are more sensitive to Ge-OH than are commensal species even if the nature of this selectivity remains unsettled [20]. Orally administered Ge-OH (30 and 120 mg kg^(− 1)^die) strongly improved the clinical signs of colitis and significantly reduced microbial dysbiosis and cyclooxygenase-2 (COX-2) expression in the gut wall of mice [24]. These results are in agreement with those obtained by Medicherla and co-authors [25] who found significantly reduced inflammation in the colon specimens of colitic mice after oral administration of Ge-OH (50 and 100 mg kg^(− 1)^die).

Since Ge-OH is a non-toxic compound, classified as Generally Recognized As Safe (GRAS) by the US Food and Drug Administration, and the European Food Security Agency hazard assessment conclusion for Ge-OH established a Derived No Effect Level (DNEL) of 13.5 mg kg^(− 1)^ die for humans, we conducted a pilot study on IBS patients to verify the hypothesis that the anti-inflammatory and anti-dysbiotic properties of Ge-OH (8 mg kg^(− 1)^ die) could improve the quality of life of these patients.

## Methods

### Study population

Inclusion criteria were: subjects aged 18 to 65 years, IBS diagnosis based on Rome III Criteria and BMI (kg m^(− 2)^) < 27 with a weight between 48 kg and 104 kg. Exclusion criteria were: intolerance to lactose or known food allergies, concomitant treatment with non-steroidal anti-inflammatory drugs and antibiotics, and consumption of functional food, food supplements, probiotics and prebiotics within two months prior to the screening visit. Women in pregnancy and lactation, subjects with a diagnosis of inflammatory bowel disease or celiac disease were also excluded, together with subjects with food allergy to Ge-OH and/or soya, subjects with serious concomitant diseases that, in the opinion of the investigator, contraindicate the patient’s participation in the study and also subjects in experimental drug treatment within two months prior to the screening visit. Any other inflammatory condition was excluded in these patients by C Reactive Protein (CRP) and Cell Blood Count (CBC), routinely performed as per clinical practice.

Consumption of functional food and/or food supplements (including probiotics and prebiotics) was not forbidden during the trial but it was considered a drop-out criterion. Patients were asked to maintain their normal diet during the trial. They were informed of the full nature and purpose of the study, and provided written informed consent before entering the trial. The study was conducted in conformity with the principles of Declaration of Helsinki and Good Clinical Practice. The sites involved in enrollment and data collection were the Inflammatory Bowel Disease Unit at S. Orsola-Malpighi University Hospital, Bologna, Italy and the Gastroenterology Unit at Spedali Civili di Brescia Hospital, Brescia, Italy. Biological samples were analyzed at Dept. of Biological, Geological and Environmental Sciences, University of Bologna.

The study was approved by the local Hospital Ethics Committees (Ethics Committee of the AOU Policlinico S. Orsola-Malpighi; CE code 100/2013/U/Sper. and Ethics Committee of the ASST Spedali Civili di Brescia; CE code NP2047).

### Dose selection and geraniol encapsulation

Ge-OH is a monoterpenoid insoluble in water with a DNEL of 13.5 mg kg^(− 1)^ die for humans (General Population - Hazard via oral route, corresponding to 100–120 mg kg^(− 1)^ die in mice). Our preclinical study demonstrated Ge-OH efficacy starting from 30 mg kg^(− 1)^ die with a maximum efficacy at 120 mg kg^(− 1)^ die [23], corresponding to 3–12 mg kg^(− 1)^ die in humans (allometric conversion). We therefore tested a maximum dose of 8 mg kg^(− 1)^ die, leaving a margin of safety with respect to the DNEL dose. After ingestion, Ge-OH is rapidly absorbed in the intestine and quickly reaches the blood circulation [26]. In mice, Ge-OH has maximum anti-colitis activity when delivered directly to the colon [24]. For these reasons, we proceeded with the microencapsulation of Ge-OH into soy lecithin micelle suspensions (Patent n° WO 201 1/128597 Al) before filling Licaps® capsules (Capsugel, Ploermel France) containing 150 mg of microencapsulated Ge-OH each. This formulation led to Ge-OH retention in the gut, with an absolute bioavailability reduced to 50% [26].

### Trial design

**Fig. 1:**
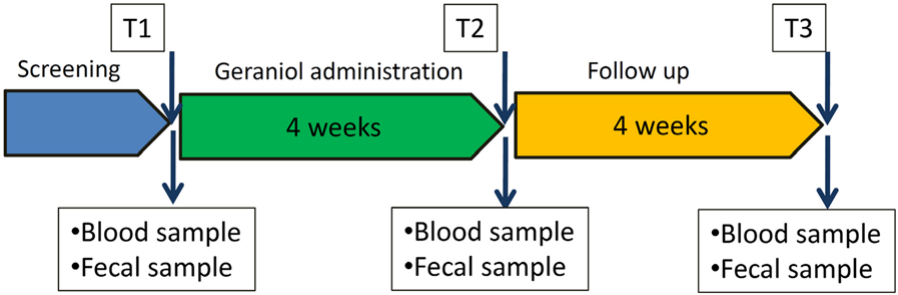
Flowchart of study protocol

The study was an interventional prospective multicentric explorative non-controlled open label trial. The trial has been retrospectively registered (registration n°: ISRCTN47041881). All subjects who met the eligible criteria received a four-week treatment with Ge-OH administered in 150-mg capsules following the dosage reported in Table 1. The treatment was followed by a four-week follow-up (Fig. 1). Study visits were scheduled at screening, at the start of treatment (V1 at T1), after four weeks of treatment (V2 at T2) and after four weeks of follow-up (V3 at T3). Clinical evaluation, physical examination, vital signs, and concomitant medications were recorded at each visit. Blood and fecal samples were collected at all visits, together with the Visual Analogue Scale for Irritable Bowel Syndrome (VAS-IBS) questionnaire completed by subjects as described below.

**Table 1:** Ge-OH administration was twice daily after meals according to the scheme below, based on the subject’s weight for a maximum dose of 10 mg Kg^(− 1)^ die

Weight	Dose
48–59 Kg	3 cp
60–74 Kg	4 cp
75–89 Kg	5 cp
90–104	6 cp

### VAS-IBS questionnaire and IBS scoring

At T1, T2 and T3, patients were asked to fill in the VAS-IBS questionnaire as support for clinical evaluation. The questionnaire is divided into two sections. The first consists of four questions evaluating clinical symptoms such as pain, abdominal distension, and general well-being. The total score of questions provides a value that may give an indication of disease trend as reported in Table 2. The second part of the questionnaire is a qualitative evaluation to investigate stool frequency and consistency and other disease-related symptoms. Fecal consistency was established through the Bristol stool scale.

**Table 2:** VAS-IBS total score and relative trend of IBS disease

VAS-IBS Score	IBS disease
< 75	Remission
75–175	Mild
175–300	Moderate
> 300	Severe

### Blood collection and cytokine analysis

Blood was collected from the antecubital fossa (left arm) by venipuncture in a Vacutainer® (BD Science) containing ethylenediaminetetraacetic acid at T1, T2 and T3. Blood samples (5 ml) were kept at 4 °C for 1 h, and then centrifuged at 1000 g for 15 min. Plasma was collected and stored at − 80 °C until cytokine and chemokine analyses.

Plasma cytokines were quantified in triplicate (plasma dilution 1:4) using a customized detection 13-plex panel (IL-1β, IL-2, IL-4, IL-5, IL-6, IL-8, IL-10, IL-12, IL-17A, IFN-γ, MCP-1, MIP-1β, TNF-α) purchased from BioRad (USA). The assays were performed in 96-well filter plates by multiplexed Luminex®-based immunoassay following the manufacturer’s instructions. IL-1β, IL-5, IL-8, IL-10, IL-17A, IFN-γ and TNF-α were read in the Luminex® using the high sensitivity mode. Microsphere magnetic beads coated with monoclonal antibodies against the different target analytes were added to the wells. After 30-min incubation, the wells were washed and biotinylated secondary antibodies were added. After further incubation for 30 min, beads were washed and then incubated for 10 min with streptavidin conjugated to the fluorescent protein phycoerythrin (streptavidin/phycoerythrin). After washing, the beads (a minimum of 100 per analyte) were analyzed in a BioPlex 200 instrument (BioRad). Sample concentrations were estimated from the standard curve using a fifth-order polynomial equation and expressed as pg/ml after adjusting for the dilution factor (Bio-Plex Manager software 5.0). Samples below the detection limit of the assay were recorded as zero, while samples above the upper limit of quantification of the standard curves were assigned the highest value of the curve. The intra-assay coefficients of variability averaged 12%.

### Fecal microbiota analysis

Fecal samples were collected from IBS patients at T1, T2 and T3 and stored at − 20 °C until DNA extraction. Nucleic acids were extracted from 250 mg of sample using PowerSoil® DNA Isolation Kit (MoBio Laboratories, Inc., CA, USA) according to the manufacturer’s recommendations. DNA sample quality was checked using a Nanodrop 100™ (NanoDrop Technologies, Wilmington, DE, USA). The hypervariable region of the 16S rRNA gene was amplified using the universal forward primer 16S27F and reverse primer r1492, and then sequenced on a 454 GS FLX Titanium and FLX+ (Roche, Basel, Switzerland) sequencing system, at MR DNA (Molecular Research LP, Shallowater, TX, USA). Sequencing reads were deposited in SOURCEFORGE (https://sourceforge.net/projects/geraniol-in-ibs/).

Publicly available 16S rRNA gene sequence data from 12 Italian Caucasian subjects (mean age, 33 years; 7 females and 5 males) were retrieved (NCBI Sequence Read Archive, BioProject ID PRJNA340060) and used as a control to characterize microbial dysbiosis in IBS [27]. These subjects were healthy at the time of fecal sample collection, and they had no history of major gastrointestinal disorders All these subjects had not received antibiotics, probiotics or prebiotics for at least three months before sampling.

Fecal specimens from healthy controls were collected and processed in the same way. All sequence data were processed by using comparable bioinformatics pipelines [28]. Briefly, quality-filtered reads were clustered into OTUs at 97% similarity threshold using UCLUST [29]. Singleton OTUs and chimeras were removed. Taxonomy assignment was performed using the RDP classifier and BLASTn against the Greengenes database. Alpha diversity was computed using the Simpson index. Beta diversity was estimated by calculating Euclidean distances between genus-level microbial profiles.

### Safety assessments

Treatment-emergent adverse events were monitored throughout the study. The relation between adverse events and the study compound was classified by the investigators as (i) definitely related, (ii) probably related, (iii) possibly related, (iv) unknown or unable to determine, (v) probably not related, and (vi) definitely not related. The first four categories were considered study drug-related adverse events. A physical examination was performed at each visit. Laboratory tests (hematology/biochemistry/urinalysis) were planned in case of adverse events.

### Statistical analysis

Being a pilot study, the number of patients to be enrolled was calculated based on similar published studies [30]. Continuous variables are expressed as mean ± SEM of at least three independent determinations. Once the homogeneity of variances (homoscedasticity, F test) had been verified, statistical differences between groups were determined by Student’s T test using GraphPad Prism 6 (GraphPad Software Inc., CA, USA). Differences were considered statistically significant at *P* < 0.05. Categorical variables are expressed in total counts and % of counts, and were compared using χ^2^ test. Differences were considered statistically significant at *P* < 0.05. All microbiota statistical analyses, including principal component analysis (PCA) of Euclidean distances between genus-level profiles, permutation tests with pseudo-F ratios (to assess the significance of data separation in PCA space) and non-parametric tests (Wilcoxon test, paired or unpaired as needed, for alpha and beta diversity, and relative abundances of bacterial taxa), were performed in R 3.3.2 using R studio 1.0.136. In cytokine multiple comparisons, statistical analysis was performed by correcting *P* values by using the Benjamini-Hochberg method. A corrected *P* value < 0.05 was considered statistically significant. We did not perform statistical analysis, nor evaluate Simpson index or Euclidean distances in the IBS-C subtype group due to the small sample size (*n* = 3).

## Results

### Patients enrolled and clinical evaluation

**Fig. 2:**
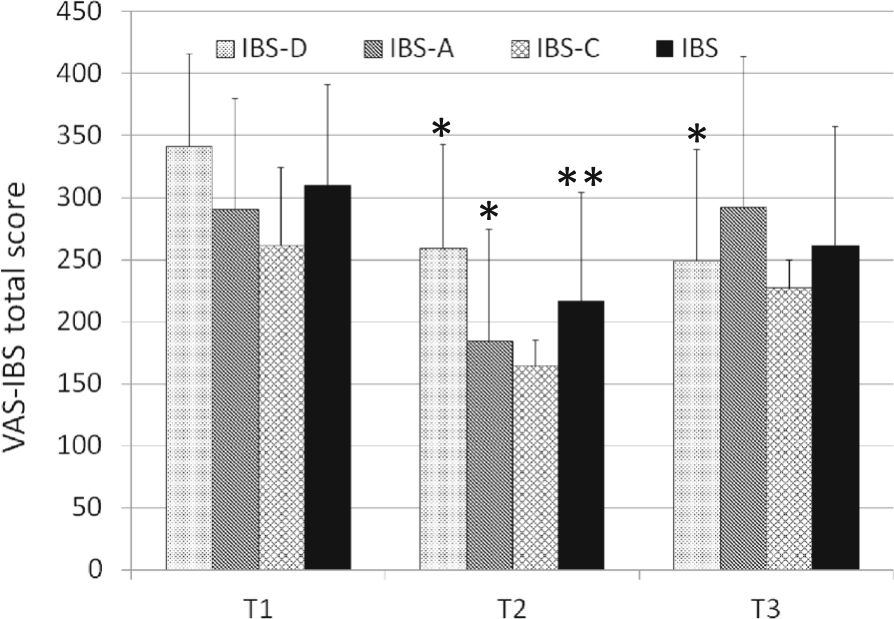
Total score of the Visual Analogue Scale for Irritable Bowel Syndrome (VAS-IBS) questionnaire administered at T1, T2 and T3. Data for the whole IBS cohort and for IBS subtypes are expressed as mean ± SD. *P < 0.05 if compared to T1; ** *P* < 0.01 if compared to T1 (Student’s T test)

The cohort of 19 IBS Italian patients (Caucasian, 8 male, 11 female, mean age, 38.84 years) included nine IBS-D, seven IBS-A and three IBS-C. None of them was post-infective IBS. None of them was following a particular diet for IBS (i.e. low carb, low gluten, low FODMAPs). No concomitant nor additional therapies, related to IBS management, were taken by patients from T1 to T3. As reported by treated patients and recorded by VAS-IBS score, individual symptoms significantly improved during the treatment period in all IBS subtypes. Improvements in bloating and intestinal regularity were declared by almost all patients. VAS-IBS score significantly decreased after treatment from a severe mean condition (309.95 ± 81.23) to a moderate mean condition (216.47 ± 87.26) (Fig. 2). This decrease was extremely statistically significant (*P* < 0.001). After four weeks of follow-up, the VAS-IBS score reached a higher mean value, still remaining in the moderate mean condition (261.42 ± 95.86) but losing statistical significance compared to T1 (*P* = 0.507). Analyzing the different IBS subtypes, at T2 both IBS-D and IBS-A subtypes showed decreased scores (258.78 ± 84.25 and 184.43 ± 89.97, respectively) that were statistically significant (*P* = 0.0421 and *P* = 0.0472, respectively), while only the IBS-D subtype maintained a VAS-IBS score significantly decreased at T3 (248.66 ± 89.92; *P* = 0.030).

### Cytokines and inflammatory markers

**Fig. 3:**
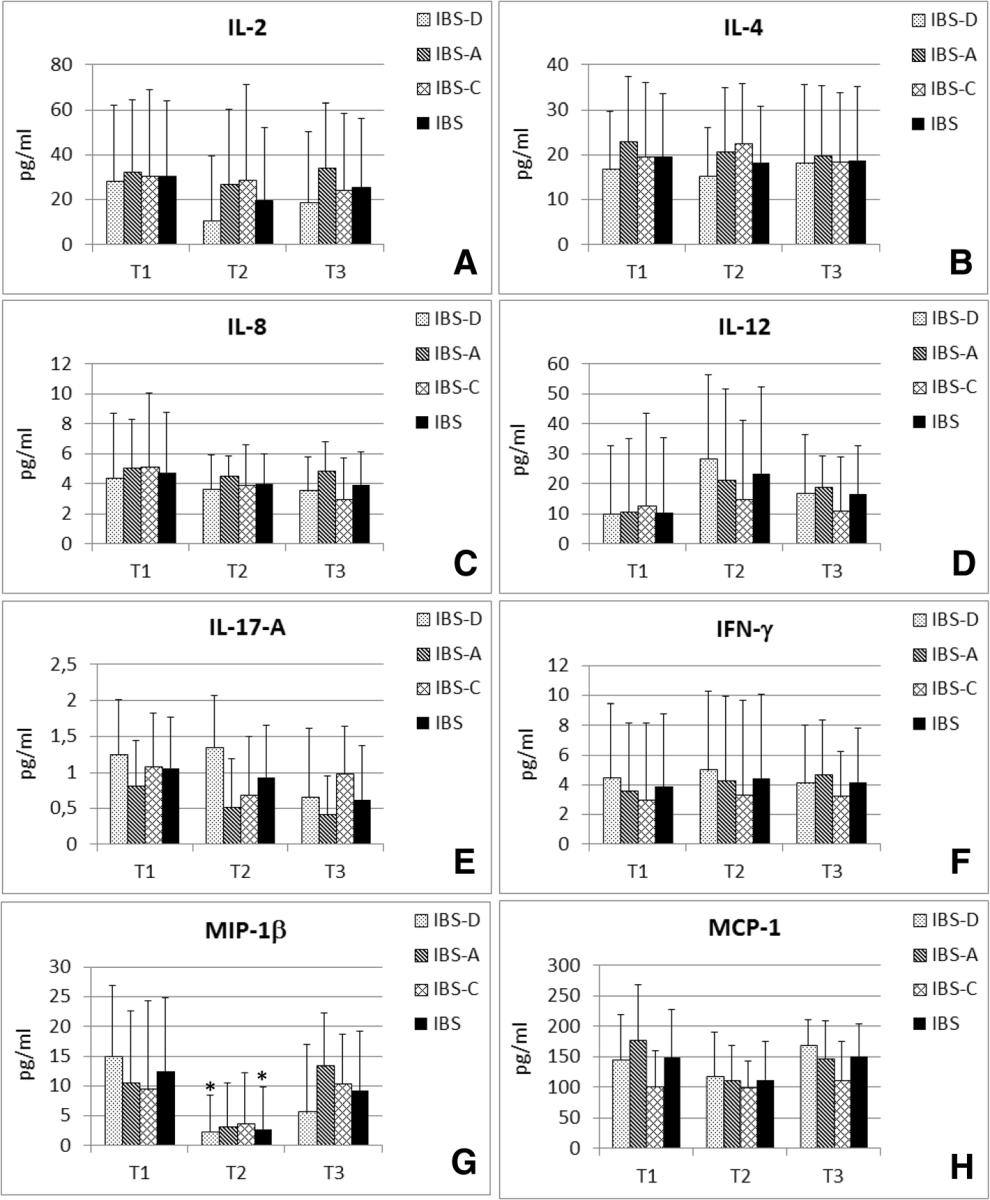
Plasma cytokine variations measured at T1, T2 and T3. Cytokines were determined using a 13-plex mouse bead immunoassay kit. Levels of IL-2 (**a**), IL-4 (**b**), IL-8 (**c**), IL-12 (**d**), IL-17A (**e**), IFN-γ (**f**), MIP-1β (**g**) and MCP-1 (**h**) are shown for the whole IBS cohort and for IBS subtypes. Data are expressed as mean ± SD of at least three replicates. Calculated *P* values were corrected for multiple comparisons by using the Benjamini-Hochberg method. *P < 0.05 if compared to T1

Among all circulating cytokines and chemokines evaluated, IL-1β, IL-5, IL-10 and TNF-α showed undetectable values in most patients. At T2, only MIP-1β (Fig. 3) showed a statistically significant reduction (*P* = 0.016), while the MCP-1, IL-6 and IL-17A decreases at T2 were only close to achieving statistical significance (*P* = 0.054, *P* = 0.059 and *P* = 0.061, respectively). Moreover, IL-6 plasma concentrations were detectable (> 0.5 pg/ml) only in 10 patients out of 19. At T3, all cytokine values were similar to those measured at T1, demonstrating that the overall systemic anti-inflammatory effect disappeared four weeks after treatment discontinuation. The only exception was IL-17A, which decreased with respect to T2 but without reaching statistical significance (*P* = 0.057). Statistical analysis performed on IBS subtypes did not show any other significant differences. At T2, MIP-1β was significantly decreased only in IBS-D subtype group (*P* = 0.032), even if this chemokine showed an evident decrease in all IBS subtypes.

### Gut microbiota modulation

**Fig. 4:**
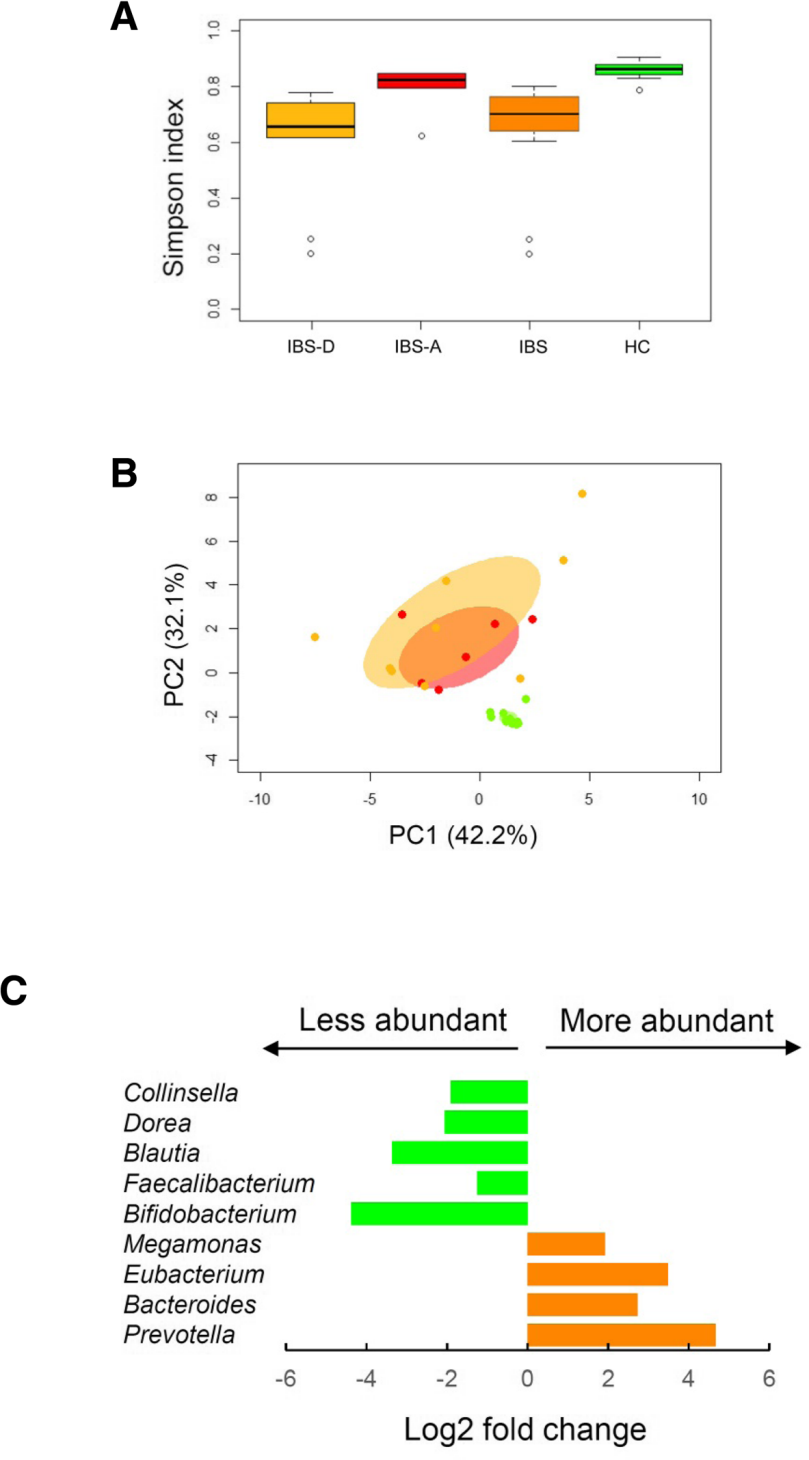
Gut microbiota signatures in IBS. **a** Box plots showing the distribution of Simpson diversity values in IBS patients (IBS-A, red; IBS-D, orange; IBS-C, black) and healthy controls (HC, green). A significant difference between IBS patients and HC was found (Wilcoxon test, *P* = 9 × 10^− 6^). **b** Principal component analysis of Euclidean distances between the genus-level intestinal microbial profiles (same color code as in A). Ellipses include 99% confidence area based on the standard error of the weighted average of sample coordinates. A significant separation between IBS and HC samples was found (permutation test with pseudo-F ratios, *P* = 2 × 10^− 5^). **c** Genus-level microbial signatures of IBS, shown as Log2-fold changes between IBS and control samples. Orange, taxa more abundant in IBS; green, taxa more abundant in HC. Wilcoxon test, *P* < 0.05

Consistent with the available literature repeatedly reporting reduced biodiversity of the intestinal microbiota in most human diseases [31], the Simpson index value was found to be significantly lower in our IBS cohort if compared to healthy controls (HC) (*P* = 9 × 10^− 6^) (Fig. 4a). Moreover, based on our findings, the gut microbiota of IBS-D patients was less biodiverse than that of IBS-A patients (*P* = 0.010). Similarly, the Euclidean distance ordination showed separation between our IBS patients and HC (permutation test with pseudo-F ratios, *P* = 2 × 10^− 5^) (Fig. 4b), with IBS characterized by a far greater interpersonal variation in microbiota structure (mean Euclidean distance ± SEM, IBS vs HC, 55.15 ± 2.23 vs 17.34 ± 0.50; Wilcoxon test, P = 9 × 10^− 28^). IBS-A and IBS-D samples were largely overlapping, with a trend towards greater interpersonal microbiota variation in the IBS-D subtype group (Fig. 4b). Genus-level taxonomic comparisons uncovered IBS-specific microbial signatures, including a considerable enrichment in the Bacteroidetes members *Prevotella* and *Bacteroides*, and in *Eubacterium* and *Megamonas*, and a low representation of commonly considered health-associated microbiota members such as *Bifidobacterium*, *Faecalibacterium*, *Blautia* and *Dorea*, as well as *Collinsella* (*P* < 0.05) (Fig. 4c). In addition, the genera *Escherichia* (mean relative abundance in IBS, 1.08%) and *Alistipes* (2.74%) were only detected in the fecal microbial communities of our IBS cohort.

**Fig. 5:**
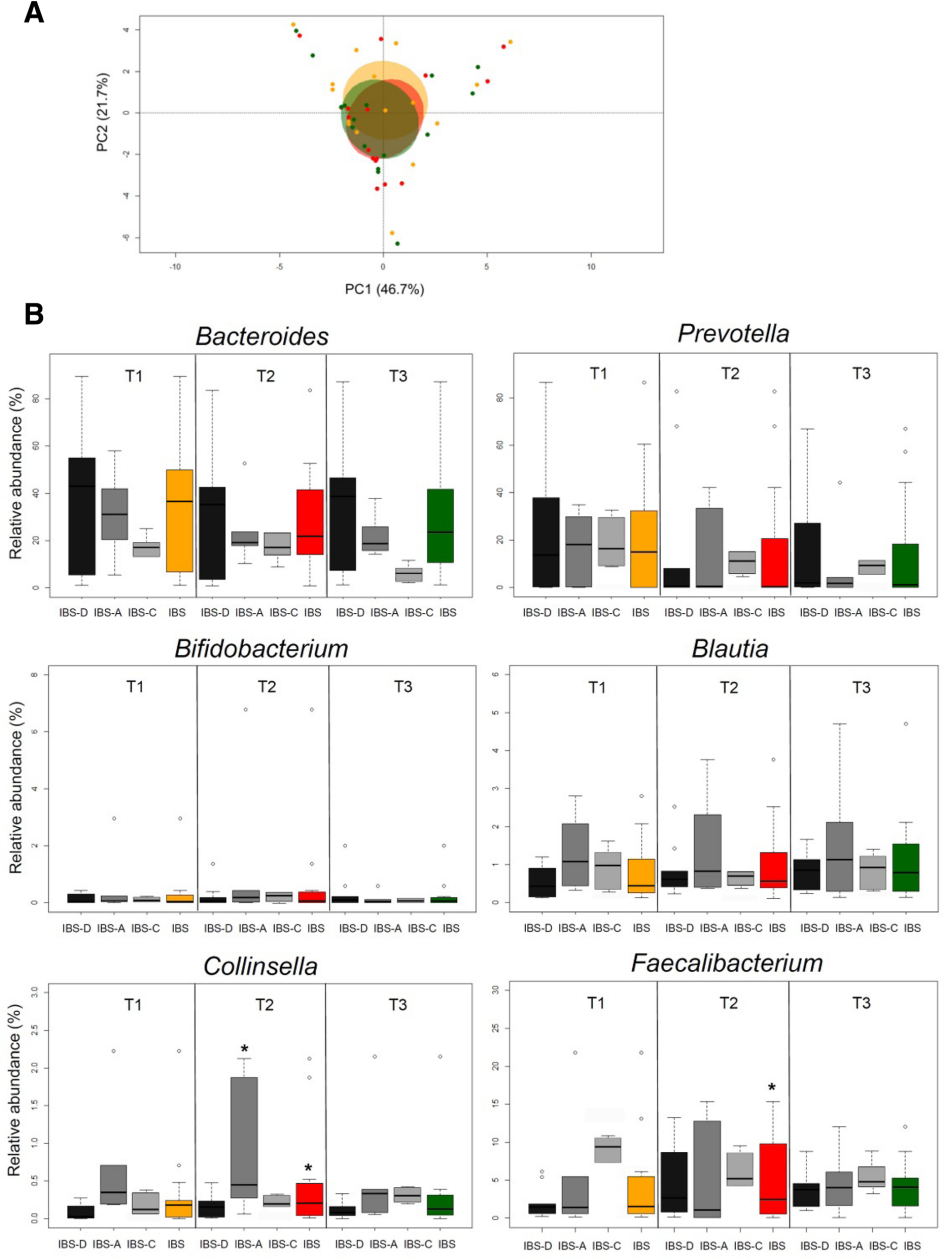
Impact of geraniol-based intervention on the intestinal microbiota structure in IBS patients. **a** Principal component analysis of Euclidean distances between the genus-level intestinal microbial profiles of IBS patients at the baseline (orange), after four-week intervention (red), and at follow-up after a further four weeks (olive green). Ellipses include the 99% confidence area based on the standard error of the weighted average of sample coordinates. A trend towards decreasing PC2 values after the intervention was observed (Wilcoxon test, *P* = 0.14). **b** Box plots showing the distribution of the relative abundance values of IBS discriminant taxa over time (T1, baseline; T2, after four-week intervention; T3, at follow-up). Data are shown for the whole IBS cohort and for IBS subtypes. The increase in the relative abundance of *Collinsella* and *Faecalibacterium* at T2 compared to the baseline (T1) was statistically significant (*, Wilcoxon test, *P* = 0.04)

After four-week GeOH administration, the gut microbiota biodiversity in IBS patients tended to increase, even if the difference was not statistically significant (Simpson index at T2, mean ± SEM, 0.69 ± 0.05; *P* = 0.4). Likewise, a slight separation between T1 and T2 samples was apparent in the PCA plot, with a shift towards decreasing PC2 values, but statistical significance was not achieved (mean PC2 coordinate ± SEM, T1 vs T2, 0.60 ± 0.62 vs − 0.30 ± 0.63; *P* = 0.14) (Fig. 5a). At the compositional level, the gut microbiota of Ge-OH-receiving IBS patients showed a significant increase in the relative abundance of *Collinsella* and *Faecalibacterium* at T2 compared to the baseline (for both, *P* = 0.04; Fig. 5b), while no differences were observed for *Escherichia* and *Alistipes*. the relative abundances of *Collinsella* was found significantly increased (*P* = 0.03) at T2 in IBS-A subtype, while no other significance was observed in the different IBS subtype groups. Although not significant, trends towards increased proportions of *Bifidobacterium*, *Blautia* and *Faecalibacterium*, and decreased percentages of *Bacteroides* and *Prevotella* were also observed (*P* ≤ 0.2) (Fig. 5b).

At follow-up after a further four weeks, the alpha diversity value was still comparable to that detected after the intervention (Simpson index at T3, mean ± SEM, 0.70 ± 0.05). Similarly, follow-up samples generally overlapped with samples at T2 in the PCA plot, still showing lower PC2 coordinates compared to the baseline (mean ± SEM, − 0.30 ± 0.63) (Fig. 5a). No difference was observed in the genus-level compositional structure of the gut microbiota between T2 and T3 samples. Compared to the baseline, the trends towards increased percentages of *Blautia* and decreased proportions of *Prevotella* were still maintained (*P* ≤ 0.3) (Fig. 5b).

### Safety and tolerability

No adverse events related to Ge-OH administration were reported during the study. Ge-OH orally administered using soy lecithin micelles was well-tolerated by patients.

## Discussion

Ge-OH is a natural monoterpene classified in the GRAS category [32]. Its therapeutic potential includes anti-inflammatory, antioxidant, and antibacterial effects, often evidenced following oral administration in rodents of doses ranging from 50 mg kg^(− 1)^ die to more than 200 mg kg^(− 1)^ die [20, 23, 33]. Nevertheless, to the best of our knowledge, this pilot study was the first to administer geraniol orally to humans. The potential therapeutic use of geraniol to target gut dysbiosis and inflammation appears promising, but in its free form, Ge-OH is easily absorbed in the small intestine and does not reach the colon except in very small quantities [26]. Therefore, Ge-OH must be associated with vehicles capable of maximizing its delivery into the colon.

This study evaluated the capacity of Ge-OH delivered to the large intestine in soy lecithin micelles to reduce symptomatology in IBS patients by modulating the gut microbiota known to be strongly altered in this pathology [13]. In particular, Ge-OH treatment resulted in increased relative abundances of *Collinsella* and *Faecalibacterium*. Interestingly, a reduction in the amount of *Collinsella aerofaciens* was previously observed in the fecal microbiota of IBS patients compared with healthy controls, and the decreased abundance of *Collinsella* has been associated with the severity of IBS symptoms [34]. On the other hand, the well-known short-chain fatty acid producer *Faecalibacterium* is among the most representative species found to be decreased in IBS and many other intestinal and metabolic diseases [35]. Since no patient before and during the trial used a particular diet we can exclude a “diet effect” on symptomatology improvement and microbiota modifications.

Ge-OH administration also led to increased ecosystem biodiversity in IBS patients. Even if this modulation was not statistically significant, probably due to the small number of patients enrolled and large inter-individual differences, the increased diversity was still detectable at T3, suggesting a lasting Ge-OH effect on microbiota structure. This effect is not in contrast with Ge-OH antimicrobial activity, since it is well established that its IC50 values vary a lot between different bacterial genera and species [20]. Indeed, genera such as *Bacteroides* and *Prevotella*, found to be enriched in IBS, decreased their relative abundance following Ge-OH treatment, even if statistical significance was not achieved. It is noteworthy that a similar pilot study in 19 IBS patients reported that the IBS-associated imbalance of the intestinal microbiota was not reverted by VLS#3 probiotic supplementation [30].

The pathogenesis of IBS has also been linked to a low grade of gut and systemic inflammation [36]. Furthermore, levels of chemotactic chemokines, such as monocyte chemoattractant protein-1 (MCP-1/CCL2), macrophage inflammatory protein-1β (MIP-1β/CCL4) and CXCL16, were found to be higher in the sera and stools of idiopathic IBS patients [8]. Overall, it is reasonable that a sort of self-sustaining inflammatory loop between the gut microbiota and low-grade gastrointestinal inflammation exists in IBS patients. Our results show that Ge-OH treatment is capable of significantly reducing serum MIP-1β at T2. MCP-1, IL-6 and IL-17A were also modulated by Ge-OH at T2, but their decrease was only close to reaching statistical relevance. These antinflammatory effects may be driven by the more protective profile of the intestinal community induced by Ge-OH modulation of gut bacteria. Data obtained in this trial are consistent with our previous experiments in mice, where Ge-OH oral administration using soy lecithin micelles resulted in a consistent decrease in pro-inflammatory circulating cytokines associated with a positive microbiota modulation in dextran sulfate sodium (DSS)-induced colitis [24]. This effect was particularly strong when Ge-OH was administered directly into the colon by enema. In rat, soy lecithin micelles deliver only 50% of orally administered Ge-OH to the large bowel since 50% is adsorbed in the small intestine [26]. It is therefore presumable that in this formulation only an estimated 4 mg kg^(− 1)^ die of geraniol effectively reached the colon of our IBS patients.

We are conscious that, since the VAS-IBS score is based on patients’ self-reported symptoms, it cannot quantify the placebo effect that in this particular pathology is known to range between 37 to 47% for pharmacological treatments and complementary medicine [37]. Despite this, the fecal microbiota analysis showed that Ge-OH is able to partially revert dysbiosis in this cohort of patients. We are aware that our study has important limitations, namely the absence of a double-blind placebo arm. Moreover, the number of patients enrolled is small. On the other hand, we carefully selected patients, and the results in terms of symptomatology, reduced dysbiosis and decreased pro-inflammatory chemokines are certainly promising, especially for IBS-D subtypes, in which Ge-OH effect on symptomatology seems to be maintained four weeks after taking the last dose of this monoterpene.

## Conclusions

The data obtained from this study are promising and statistically significant in terms of microbiota modulation, decrease of circulating MIP-1β and reduced VAS-IBS score. A placebo-controlled study on a larger population is now needed to confirm the effectiveness of Ge-OH in improving the symptomatology of IBS patients. Nevertheless, food supplement formulations enriched in Ge-OH and capable of delivering it to the large bowel could be used to counteract or prevent dysbiosis.

## Abbreviations

DNEL: Derived No Effect Level
FODMAPs: Fermentable Oligo-, Di- and Mono- saccharides And Polyols
Ge-OH: Geraniol
GRAS: Generally Recognized As Safe
IBS: Irritable Bowel Syndrome
VAS-IBS: Visual Analogue Scale for Irritable Bowel Syndrome
